# Comparison of Heat and Drought Stress Responses among Twelve Tartary Buckwheat (*Fagopyrum tataricum*) Varieties

**DOI:** 10.3390/plants11111517

**Published:** 2022-06-06

**Authors:** Lauranne Aubert, Muriel Quinet

**Affiliations:** Earth and Life Institute—Agronomy, Université Catholique de Louvain, B-1348 Louvain-la-Neuve, Belgium; lauranne.aubert@uclouvain.be

**Keywords:** abiotic stress, antioxidant, plant growth, plant reproduction, physiological parameters, yield parameters

## Abstract

The use of orphan crops could mitigate the effects of climate change and improve the quality of food security. We compared the effects of drought, high temperature, and their combination in 12 varieties of Tartary buckwheat (*Fagopyrum tataricum*). Plants were grown at 21/19 °C or 28/26 °C under well-watered and water-stressed conditions. Plants were more discriminated according to environmental conditions than variety, with the exception of Islek that was smaller and produced fewer leaves, inflorescences, and seeds than the other varieties. The combination of high temperature and water stress had a stronger negative impact than each stress applied separately. The temperature increase stimulated leaf and flower production while water stress decreased plant height. Leaf area decreased with both temperature and water stress. High temperature hastened the seed initiation but negatively affected seed development such that almost all seeds aborted at 28 °C. At 21 °C, water stress significantly decreased the seed production per plant. At the physiological level, water stress increased the chlorophyll content and temperature increased the transpiration rate under well-watered conditions. High temperature also increased the polyphenol and flavonoid concentrations, mainly in the inflorescences. Altogether, our results showed that water stress and temperature increase in particular negatively affected seed production in *F. tataricum.*

## 1. Introduction

Earth’s global surface temperature has increased by around 1.1 °C compared with the average in 1850–1900, and it is expected to further warm up by 2.5 to 4 °C over the next 100 years [1]. Higher temperatures are often accompanied by drought [2,3,4]. Organisms are, thus, brought to know more and more periods of heat and drought. As sessile organisms, plants cannot move in search of a better habitat in the face of unfavorable conditions and need to develop mechanisms to survive [2,3,5,6]. High temperature and drought are known to have many negative impacts on plants at the morphological, biochemical, and physiological levels [3,7,8]. In the interest of agriculture, the impact of environmental disruptions on grain yield has often been studied [9,10,11]. High temperature tends to decrease the number of seeds produced, while drought additionally causes a reduction in seed size [10,12,13]. High temperature and drought also cause oxidative stress [3,14]. However, under field conditions, high temperature and drought usually occur simultaneously [5,10]. The consequences of the combination of these two disturbances are not limited to the addition of their separate effects [2,3,5,15]. Studies investigating the influence of their combination on crop development remain nevertheless limited [5,10]. Fortunately, over the centuries, adaptations have been selected and allow many species to cope with poor conditions [7,8,10]. For example, to deal with oxidative stress, antioxidants are produced to protect the plant [10,14]. However, crop species remain sensitive to abiotic modifications compared to wild species. Studying the effects of combined environmental disruptions on the growth and yield of cultivated plants is, therefore, important to identify promising ways of improvement for breeding programs [5].

Presently, one of the main strategies for sustainable agriculture is to release the genetic potential of underutilized crops [16,17,18]. Broadening our food sources through the integration of so-called orphan crops, such as pseudocereals, can help to mitigate the effects of environmental change and improve qualitative food security. Pseudocereals are considered as rich foods because of their protein quality and content, high mineral content, and healthy and balanced food quality [17,18,19]. They are also gluten-free [16], making them interesting for people with celiac disease. Moreover, they are tolerant to various stress factors such as drought and heat [16]. However, despite their potential, efforts toward the genetic improvement of pseudocereals lag considerably behind those of major crops [20,21]. Improvement of these crops requires a better understanding of their physiology.

Buckwheat is a pseudocereal originating from China; domestication most probably occurred around 5000–6000 BCE, and it became a pan-Eurasian crop by 3000 BCE [22]. This crop has gained considerable interest worldwide due to its nutritional, economical, and pharmaceutical values [18]. Buckwheat is mainly consumed as flour or groats, but it is also used to produce noodles, porridge, bread, pancakes, sprouts, and even drinks [23,24]. Buckwheat is considered to have high nutritional value and medicinal qualities, and its benefits have been highlighted in several reviews [23,24,25,26,27,28,29,30,31]. Two buckwheat species are cultivated, common buckwheat (*Fagopyrum esculentum* Moench) and Tartary buckwheat (*Fagopyrum tataricum* (L.) Gaertn), although the first accounts for 90% of the production [19]. *Fagopyrum tataricum* is homostylous and self-pollinated and has higher yield and flavonoids than the self-incompatible *F. esculentum* [19,28,32,33,34]. Research about *F. tataricum* is increasing (e.g., [35,36,37,38,39,40]), and the whole-genome resequencing of 510 *F. tataricum* germplasms has allowed the identification of several genomic loci that contribute to the formation of important quality and yield traits in this species [34]. Genome analyses also isolated genes predicted to be involved in abiotic stress response such as aluminum, cold, or drought stress [41,42]. Although *F. tataricum* was reported to be widely adaptable to hostile environments [43], its physiology and response to abiotic stress remain largely unknown. In previous studies, we compared the response of *F. esculentum* and *F. tataricum* to high temperature [44] and water stress [45]. High temperature mainly affected the reproductive stage and increased the antioxidant concentration in the two buckwheat species [44]. However, both species differed in their strategy to cope with water stress [45]. Vegetative and reproductive growth was less affected in *F. tataricum* than in *F. esculentum*, and it was suggested that *F. tataricum* exhibited traits of drought tolerance [45]. However, these studies investigated only one variety per species [44,45], and it is known that the response to abiotic stress may differ among varieties inside the same species [43,46].

In this study, we compared the response of 12 *F. tataricum* varieties to high temperature, water stress, and their combination in order to identify tolerant varieties and to understand the underlying physiological response mechanisms to combined stress.

## 2. Results

Plants of 12 varieties of *F. tataricum* (Table S1) were grown in a greenhouse under two temperature and two watering conditions for 10 weeks, starting 2 weeks after sowing. Plants were grown at either 21 °C/19 °C (day/night) under well-watered (21WW) and water-stressed (21WS) conditions or at 28 °C/26 °C (day/night) under well-watered (28WW) and water-stressed (28WS) conditions (Figure S1).

**Figure 1:**
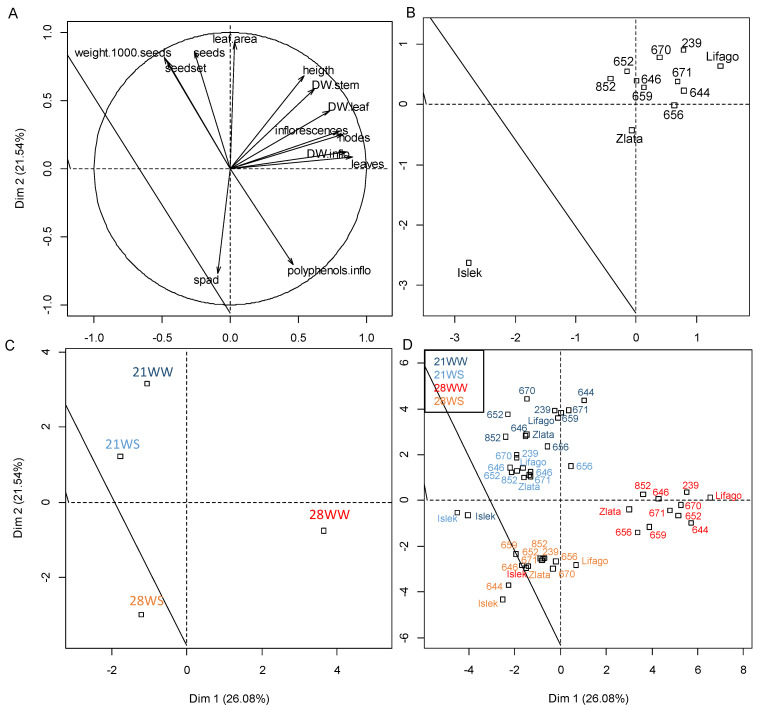
Principal component analysis (PCA) of plant growth and physiological parameters in *F. tataricum* varieties subjected to two temperatures (21 °C vs. 28 °C) and water supply conditions (well-watered vs. water-stressed). (**A**) Variable graph of PCA presenting growth and physiological parameters; only parameters with cos^2^ > 0.5 are shown. (**B**) Individual graph presenting the average individuals according to the varieties of *F. tataricum*. (**C**) Individual graph presenting the average individuals according to the treatments: 21WW: 21 °C well-watered, 21WS: 21 °C water stress, 28WW: 28 °C well-watered, 28WS: 28 °C water stress. (**D**) Individual graph presenting the varieties according to the treatments. Dim 1 and Dim 2: dimensions 1 and 2 of the PCA; DW: dry weight; inflo: inflorescence; 659: PI481659; 656: PI481656; 652: PI481652; 239: PI427239; 852: PI476852; 646: PI481646; 670: PI481670.

The principal component analysis (PCA) demonstrated that 26% of the variance was explained by axis 1 (Dim1), while 21.5% was explained by axis 2 (Dim2) (Figure 1). Axis 1 was mainly explained by plant growth parameters such as leaf and inflorescence production, the number of nodes on the main stem, and plant dry weight (DW) (Figure 1A). Axis 2 was mainly explained by the leaf area and the yield parameters (number of seeds, seed set, and 1000-seed weight), as well as by the chlorophyll content in the leaves and the polyphenol concentration in the inflorescences (Figure 1A). Plants were more discriminated according to treatment than variety (Figure 1B–D). Most of the varieties grouped together with the exception of Islek that produced smaller plants with fewer leaves, inflorescences, and seeds (Figure 1B,D). Axis 2 discriminated the plants according to temperature, with plants grown at 21 °C showing higher leaf area, seed production, and seed weight, and plants grown at 28 °C showing higher leaf chlorophyll concentration and polyphenol concentration in the inflorescences. Axis 1 separated the 28WW treatment from the other treatments, showing that plants produced more leaves and inflorescences under the 28WW condition.

### 2.1. Vegetative Growth

Leaf production, leaf area, plant height, and the number of ramifications per plant differed among varieties, temperatures, and watering conditions (Table 1).

**Figure 2:**
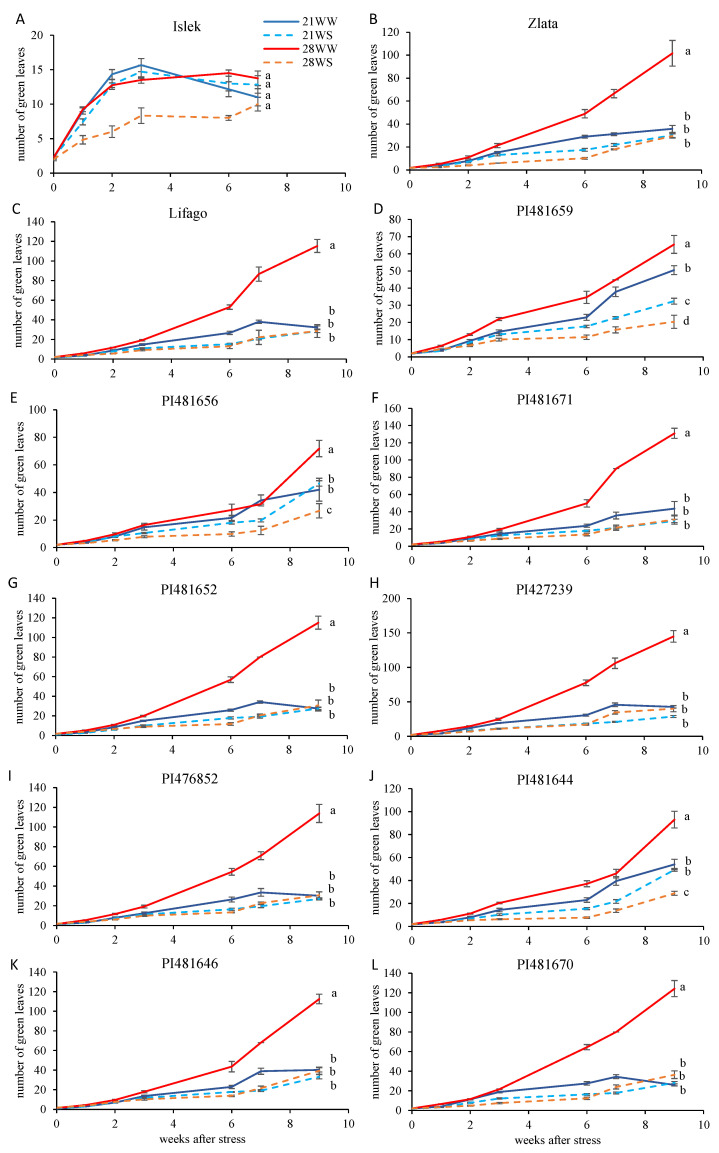
Leaf production of *F. tataricum* varieties subjected to two temperatures (21 °C vs. 28 °C) and water supply conditions (well-watered vs. water-stressed). Varieties (**A**) Islek, (**B**) Zlata, (**C**) Lifago, (**D**) PI481659, (**E**) PI481656, (**F**) PI481671, (**G**) PI481652, (**H**) PI427239, (**I**) PI476852, (**J**) PI481644, (**K**) PI481646, and (**L**) PI481670. 21WW: 21 °C well-watered, 21WS: 21 °C water stress, 28WW: 28 °C well-watered, 28WS: 28 °C water stress. Values followed by a same letter for the same variety were not statistically significant at the 5% level at the end of the experiment.

The highest mean leaf production (all conditions combined) at the end of the experiment was observed in PI427239 (64 leaves), while the lowest was observed in Islek (12 leaves) (Figure 2). Plants grown under the 28WW condition produced at least two times more leaves than under the other conditions in all varieties with the exception of Islek and PI481659 (Figure 2A,D, Table S2). There were no differences in leaf production under the other conditions (21WW, 21WS, 28WS) in Zlata, Lifago, PI481671, PI481652, PI427239, PI476852, PI481646, and PI481670 (Figure 2B,C,F–I,K,L), while PI481659, PI481656, and PI481644 produced fewer leaves under 28WS conditions than at 21 °C (Figure 2D,E,J). The leaf production under 28WS conditions was also lower than that under the other conditions in Islek during the first 6 weeks of treatment (Figure 2A). Overall, the leaf decrease between 28WW and 28WS ranged from 27% in Islek to 76% in PI481671.

**Figure 3:**
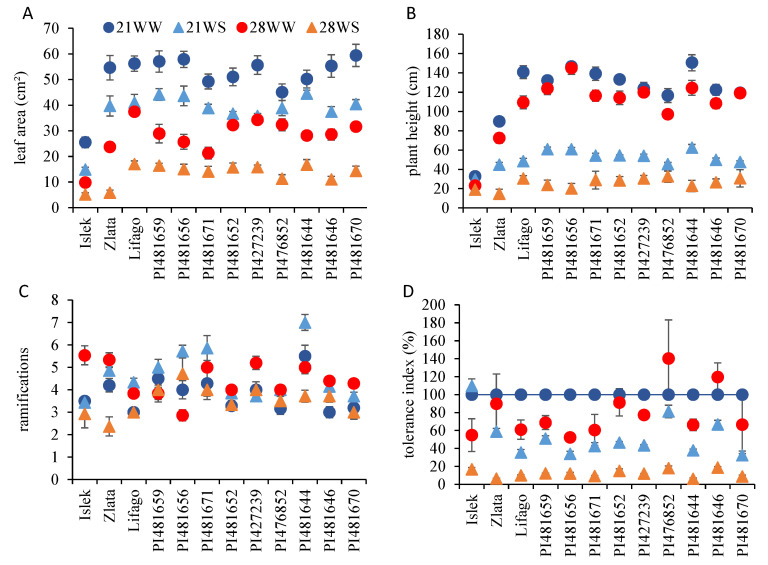
Vegetative growth parameters of *F. tataricum* varieties subjected to two temperatures (21 °C vs. 28 °C) and water supply conditions (well-watered vs. water-stressed). (**A**) Leaf area, (**B**) plant height, (**C**) number of ramifications per plant, and (**D**) tolerance index. 21WW: 21 °C well-watered, 21WS: 21 °C water stress, 28WW: 28 °C well-watered, 28WS: 28 °C water stress.

Although leaf production was stimulated under 28WW conditions, the leaf area significantly decreased with temperature and water stress (Figure 3A, Table S2). In most varieties, the highest leaf area was observed under 21WW conditions, followed by 21WS and 28WW, and the smallest leaves were observed under 28WS conditions (Figure 3A). The leaf area decrease between 21WW and 28WS ranged from 67% in PI476852 to 89% in Zlata.

On average, for all conditions combined, the tallest varieties were PI481656 (93 cm) and PI481644 (90 cm), and the smallest was Islek (26 cm) (Figure 3B, Table 1). Plant height varied with temperature and watering condition in all varieties (Table S2). Overall, plants grown under water stress conditions were two or three times smaller than plants grown under well-watered conditions (Figure 3B). Regarding environmental conditions, the tallest plants were observed under 21WW conditions, with the exception of PI481656, PI427239, and PI481670 where plants had the same height under 21WW and 28WW conditions, while the smallest plants were observed under 28WS conditions regardless of the variety (Figure 3B). The plant size decrease between the tallest and the smallest plants ranged from 42% in Islek to 86% in PI481656 (Figure 3B).

The effect of temperature and water stress on plant branching differed among varieties (Figure 3C, Table 1). The number of ramifications per plant varied with temperature in PI481656, PI427239, and PI481644, and with water stress in Islek, Zlata, PI427239, and PI481656 (Table S2). Islek and PI427239 produced more ramifications under 28WW conditions, while PI481656 produced fewer ramifications under 28 WW conditions compared to the other conditions (Figure 3C). PI481656 and PI481644 produced more ramifications under 21WS conditions, while Zlata and PI481644 produced fewer ramifications under 28WS conditions compared to the other conditions.

The tolerance index was calculated on the basis of the plant DW and varied with the variety, temperature, and watering conditions (Figure 3D, Table 1). It decreased with temperature and water stress in most varieties with the exception of Islek, where the tolerance index was above 100% under 21WS conditions, and PI476852 and PI4481646, where it was above 100% under 28WW conditions (Figure 3D, Table S2), indicating that plant growth was higher under these conditions than under 21WW conditions. In general, the tolerance index was lower under 21WS than under 28WW conditions, and it was lower than 20% under 28WS conditions in all varieties.

According to Table 1, leaf DW was mainly affected by water stress, while stem DW was affected by both temperature and water stress. The effect of temperature and water stress on leaf and stem DW also differed according to the variety (Table 1, Table 2 and Table S2). The highest total leaf DW was observed under 28WW conditions in most varieties with the exception of PI481656 where it was higher under 21WS conditions, and PI481671 and PI427239 where it was higher under 21WW conditions. The highest stem DW was also observed either under 21WW (Islek, Lifago, PI481659, PI481656, PI481671, PI481644) or 28WW (Zlata, PI481652, PI427239, PI496852, PI481646, PI481670) conditions (Table 2). The lowest leaf and stem DW was observed under 28WS conditions for all varieties (Table 2).

### 2.2. Reproductive Growth

The inflorescence production, the flowering and seeding time, the number of flowers per inflorescence, and the yield parameters (seed set, seeds per plant, and 1000-seed weight) varied according to the variety (Table 1). Regarding the environmental conditions, temperature significantly affected the inflorescence production, the node of the first inflorescence, the number of flowers per inflorescence, and the yield parameters, while the watering conditions affected the inflorescence production, the flowering and seeding dates, the number of seeds per plant, and the 1000-seed weight (Table 1).

**Figure 4:**
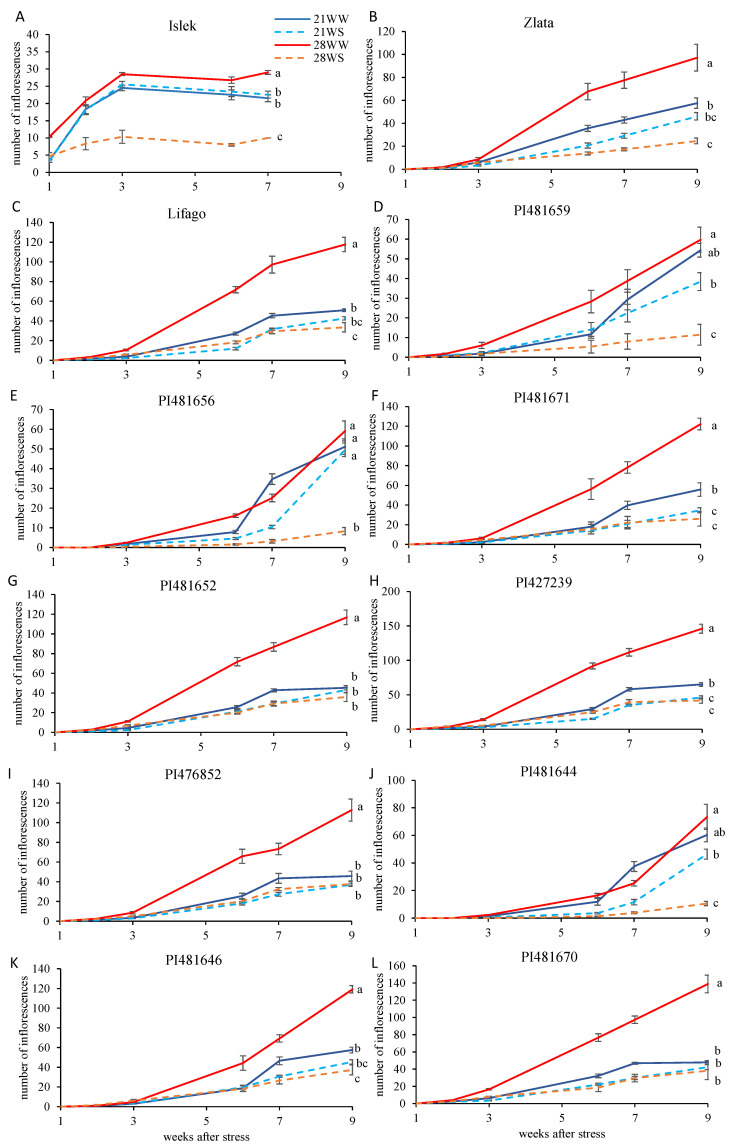
Inflorescence production of *F. tataricum* varieties subjected to two temperatures (21 °C vs. 28 °C) and water supply conditions (well-watered vs. water-stressed). Varieties (**A**) Islek, (**B**) Zlata, (**C**) Lifago, (**D**) PI481659, (**E**) PI481656, (**F**) PI481671, (**G**) PI481652, (**H**) PI427239, (**I**) PI476852, (**J**) PI481644, (**K**) PI481646, and (**L**) PI481670. 21WW: 21 °C well-watered, 21WS: 21 °C water stress, 28WW: 28 °C well-watered, 28WS: 28 °C water stress. Values followed by a same letter for the same variety were not statistically significant at the 5% level at the end of the experiment.

The highest mean inflorescence production (all conditions combined) at the end of the experiment was observed in PI427239 and PI481670 with 69 inflorescences per plant, while the lowest mean inflorescence production was observed in Islek with 22 inflorescences per plant (Figure 4). Inflorescence production was affected by both temperature and watering conditions in all varieties with the exception of Islek, Zlata, PI481659, and PI481644 where the temperature effect was not significant but differed according to the watering conditions (Figure 4, Table S2). In most varieties, the highest inflorescence production was observed under 28WW conditions, while the lowest was observed under 28WS conditions, with intermediate inflorescence production under 21WW and 21WS conditions (Figure 4). For all varieties combined, the mean number of inflorescences per plant was 99 under 28WW, 51 under 21WW, 41 under 21WS, and 27 under 28WS conditions. The decrease in inflorescence production between 28WW and 28WS ranged from 65% in Islek to 86% in PI481656 and PI481644. The difference in inflorescence production between 21WW and 21WS at the end of the experiment was only significant in PI481671 (Figure 4F). Inflorescence production was similar under 21WW, 21WS, and 28WS conditions in PI481652, PI476852, and PI481670 (Figure 4G,I,L), while it was similar under 21WS and 28WS conditions in Zlata, Lifago, PI481671, PI427239, and PI481646 (Figure 4B,C,F,H,K).

**Figure 5:**
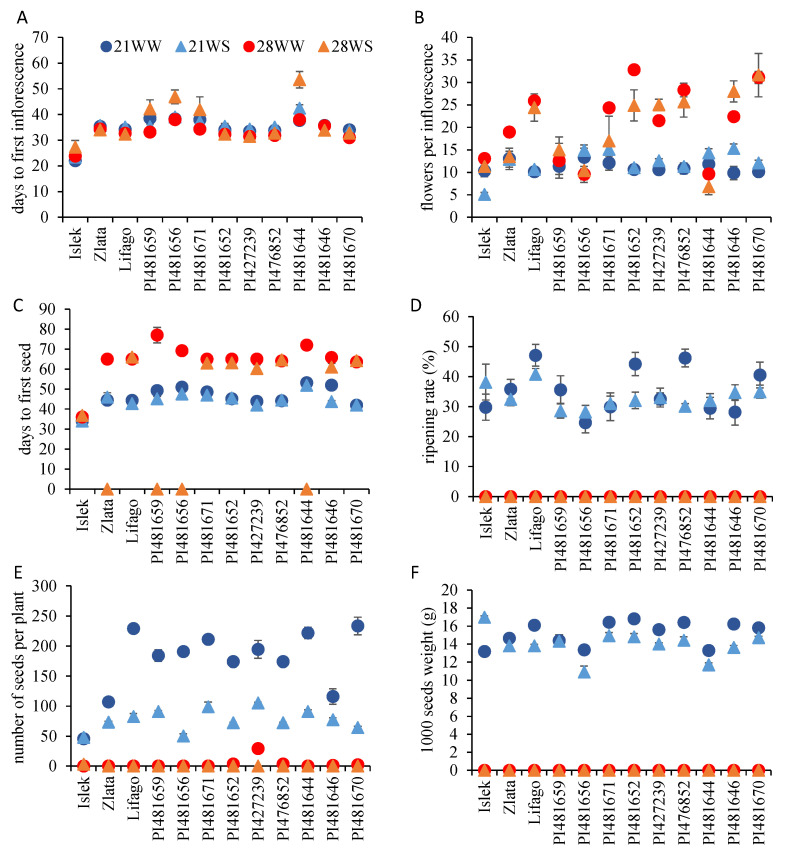
Reproductive growth parameters of *F. tataricum* varieties subjected to two temperatures (21 °C vs. 28 °C) and water supply conditions (well-watered vs. water-stressed). (**A**) Number of days from sowing to first inflorescence apparition, (**B**) total number of flowers per inflorescence, (**C**) number of days from sowing to apparition of the first green seed on the plant, (**D**) ripening rate (mature seeds per inflorescence/total number of flowers per inflorescence), (**E**) total number of normal seeds per plant, and (**F**) weight of 1000 seeds. 21WW: 21 °C well-watered, 21WS: 21 °C water stress, 28WW: 28 °C well-watered, 28WS: 28 °C water stress.

Independently of the environmental condition, Islek was the first variety to flower after a mean value (all conditions combined) of 24 days after treatment, while PI481644 was the latest to flower after a mean value of 43 days after treatment (Figure 5A). PI476852 was the first variety to flower in terms of flowering node, while PI481644 was the latest; the mean node with the first inflorescence varied from 4.7 to 7.9. Temperature and watering conditions did not affect the node of first inflorescence, with the exception of Islek and Lifago. In Islek, the node of first inflorescence increased with temperature (4.3 nodes at 21 °C vs. 4.9 nodes at 28 °C), while, in Lifago, it increased with water stress (4.6 nodes under WW vs. 5.2 under WS conditions). The number of days to flowering was affected by temperature in Zlata, Lifago, PI481652, PI427239, PI476852, and PI481670, whereas it was affected by both temperature and watering conditions in PI481656 and PI481644 (Figure 5A, Table S2). In these two last varieties, the plants grown at 28WS flowered significantly later compared to the other conditions (Figure 5A).

The mean number of flowers per inflorescence, all conditions combined, ranged from 10 in Islek to 21 in PI481670. With the exception of PI481659, the number of flowers per inflorescence varied with temperature in all varieties and with watering conditions in Islek, PI481652, and PI48646 (Figure 5B, Table S2). The number of flowers per inflorescence was generally higher at 28 °C with the exception of PI481644. Water stress decreased the number of flowers per inflorescence in Islek, which showed the lowest number of flowers per inflorescence under 21WS conditions, but increased it in PI481646, which showed the highest number of flowers per inflorescence under 28WS conditions (Figure 5B). The difference in the number of flowers per inflorescence due to environmental conditions ranged from 32% in Zlata to 68% in PI481670 and PI481652.

As for the flowering time, the first variety to produce a seed was Islek after 35 days of treatment, while the latest was PI481644 after 56 days of treatment (all conditions combined). High temperature accelerated the seeding date in all varieties, while water stress only affected the seeding date in PI481656, PI427239, and PI481646 (Figure 5C, Table S2). Zlata, PI481659, PI481656, and PI481644 never developed seeds under 28WS conditions. Even though seed initiation took place in most varieties at 28 °C, seeds aborted during seed development at this temperature, such that all varieties produced empty or misshaped seeds at 28 °C. This explains the absence or the low production of viable brown seeds under both 28WW and 28WS conditions (Figure 5D–F). The only varieties that produced some brown seeds under 28WW conditions were PI476852, PI481652, and PI427239 with a production of 2 ± 0.6, 3.3 ± 1.3, and 29 ± 5.6 seeds per plant, respectively.

The ripening rate at 21 °C (21WW and 21WS combined) ranged from 26% in PI481656 to 44% in Lifago. It was similar under 21WW and 21WS conditions in most varieties with the exception of PI481652 and PI476852, which showed a higher ripening rate under 21WW than under 21WS conditions (Figure 5D, Table S2). However, the seed production per plant was significantly lower under 21WS than under 21WW conditions in all varieties with the exception of Islek, which produced the same number of seeds under both conditions (Figure 5E, Table S2). This reduction ranged from 32–33% in Zlata and PI481646 to 73–74% in PI481670 and PI481656. The varieties with the highest mean seed production per plant at 21 °C (21WW and 21WS combined) were PI481671 (155 seeds), Lifago (156 seeds), and PI481644 (156 seeds), while the variety with the lowest number of seeds per plant was Islek (47 seeds).

On average, PI481652 showed the highest 1000-seed weight (15.9 g), while PI481656 showed the lowest 1000-seed weight (12.1 g) at 21 °C (21WW and 21WS combined). At 21 °C, the 1000-seed weight increased with water stress in Islek and decreased with water stress in the other varieties with the exception of Zlata, PI481659, and PI481670, which showed a similar seed weight under 21WW and 21WS conditions (Figure 5F, Table S2). Taking into account the mean seed production and the 1000 seed weight at 21 °C, the highest yield was observed in PI481671 and Lifago, while the lowest was observed in Islek.

### 2.3. Physiological Parameters

**Figure 6:**
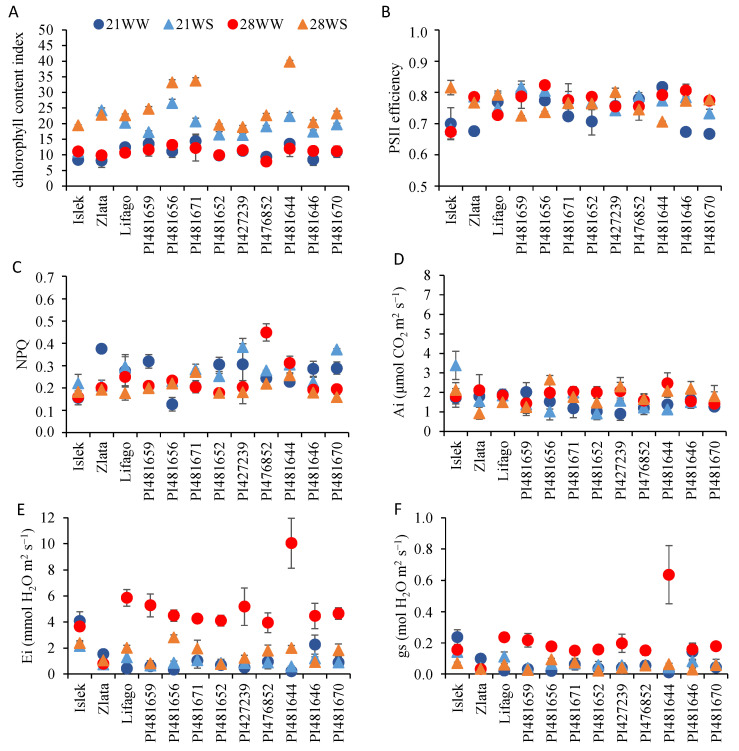
Photosynthesis and water status-related parameters of *F. tataricum* varieties subjected to two temperatures (21 °C vs. 28 °C) and water supply conditions (well-watered vs. water-stressed). (**A**) Chlorophyll content index, (**B**) photosystem 2 (PSII) efficiency, (**C**) non-photochemical quenching (NPQ), (**D**) net photosynthesis rate (Ai), (**E**) net transpiration rate (Ei), and (**F**) stomatal conductance (gs) at 8 weeks after stress imposition. 21WW: 21 °C well-watered, 21WS: 21 °C water stress, 28WW: 28 °C well-watered, 28WS: 28 °C water stress.

The chlorophyll content index (CCI) varied according to the variety and the environmental conditions (Figure 6A, Table 3). Water stress increased the CCI in all varieties, while temperature affected it in all varieties except Zlata, Lifago, PI481659, and PI476852 (Figure 6A, Table S2). In most varieties, the effect of temperature depended on the watering conditions such that plants grown under 28WS conditions showed the highest CCI, mainly in PI481656, PI481671, and PI481644, while plants grown under 28WW or 21WW conditions showed the lowest CCI (Figure 6A, Table S2).

Regarding chlorophyll fluorescence parameters, the efficiency of photosystem 2 (ϕPSII) and the nonphotochemical quenching (NPQ) did not differ among varieties and environmental conditions with the exception of NPQ, which was affected by temperature (Figure 6B,C, Table 3). Some differences were nevertheless observed at the variety level; ϕPSII decreased with temperature and water stress in PI481644 (Figure 6B, Table S2), while NPQ decreased with temperature in PI481652 and PI481670 and with temperature and water stress in Zlata (Figure 6C, Table S2).

Regarding gas exchange, the net photosynthesis rate (Ai) was similar among varieties but varied with temperature (Figure 6D, Table 3). At the variety level, Ai increased with temperature only in PI481656, PI481652, PI427239, and PI481644 (Figure 6D, Table S2). In contrast to Ai, the net transpiration rate (Ei) and the stomatal conductance (gs) varied among varieties (Table 3, Figure 6E,F). These parameters were also affected by both temperature and watering conditions, and their impact depended on the variety (Table 3). With the exception of Zlata for Ei and of PI481652 for gs, all varieties were affected by temperature and/or water stress regarding water status-related parameters (Figure 6E,F, Table S2). The Ei was significantly higher under 28WW conditions compared to the other conditions in all varieties with the exception of Islek and Zlata, while the lowest Ei values were usually observed under water stress conditions (Figure 6E). In the same way, the highest gs values were observed under 28WW conditions in all varieties with the exception of Islek and Zlata, where the highest gs value was observed under 21WW conditions (Figure 6F).

### 2.4. Antioxidant Production

**Figure 7:**
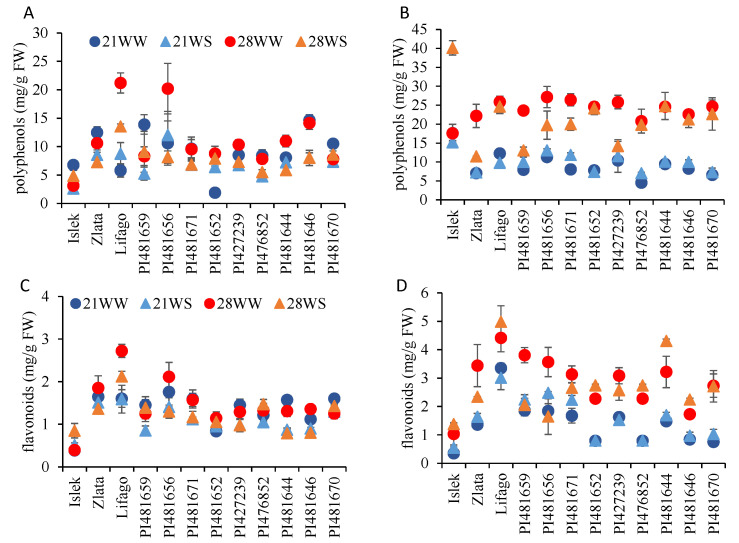
Antioxidant content of *F. tataricum* varieties subjected to two temperatures (21 °C vs. 28 °C) and water supply conditions (well-watered vs. water-stressed). (**A**,**B**) Total polyphenol concentration and (**C**,**D**) total flavonoid concentrations in leaves (**A**,**C**) and inflorescences (**B**,**D**). 21WW: 21 °C well-watered, 21WS: 21 °C water stress, 28WW: 28 °C well-watered, 28WS: 28 °C water stress.

The concentrations of polyphenols and flavonoids were significantly higher in the inflorescences compared to the leaves (Figure 7). Their concentrations varied among varieties (Table 3). The mean polyphenol concentrations (all conditions combined) ranged from 3 mg/g in Islek to 14 mg/g in PI481656 in the leaves, and from 12 mg/g in Zlata to 23 mg/g in Islek in the inflorescences. The mean flavonoid concentrations (all conditions combined) ranged from 0.5 mg/g and 0.8 mg/g in Islek to 2 mg/g and 4 mg/g in Lifago in the leaves and the inflorescences, respectively. In general, the polyphenol concentrations were affected by temperature but not by watering conditions, while the flavonoid concentrations were affected by both temperature and watering conditions (Table 3). The concentrations of polyphenols and flavonoids were more affected by the environmental conditions in the inflorescences than in the leaves (Figure 7).

At the variety level, the polyphenol concentration in the leaves was affected by temperature and watering conditions in Islek, Lifago, and PI481644 and by watering conditions only in PI427239, PI476852, and PI481646; their impact depended on the variety (Figure 7A, Table S2). The difference in leaf polyphenol concentrations among conditions ranged from 24% in PI481670 to 60% in Islek, Lifago, and PI481656. The polyphenol concentration in the inflorescences increased with temperature in all varieties while it was only affected by water stress in Islek and PI481659 (Figure 7B, Table S2). As a result, the highest concentrations of polyphenols in the inflorescences were observed either under 28WW (Zlata, PI481659, PI481656, PI481671, and PI427239) or under 28WW and 28WS (Lifago, PI481652, PI476852, PI481644, PI481646, and PI481670) conditions, with the exception of Islek, where the highest concentrations were observed under 28WS and 21WW conditions. The difference in inflorescence polyphenol concentrations among conditions ranged from 59% in PI427239 and PI481656 to 78% in PI476852.

The effects of temperature and watering conditions on the flavonoid concentrations at the variety level were almost the same as observed for polyphenols (Figure 7, Table S2). The leaf flavonoid concentration was affected by temperature in Lifago and by watering conditions in Islek, PI427239, PI481644, and PI481646 (Figure 7C, Table S2). Water stress increased leaf flavonoid concentration in Islek and decreased it in PI427239, PI481644, and PI481646 (Figure 7C). In Lifago, the highest leaf flavonoid concentration was observed under 28WW conditions (Figure 7C). The difference in leaf flavonoid concentrations among conditions ranged from 22% in PI481670 to 50% in PI481644 and Islek. The flavonoid concentrations in the inflorescences increased with temperature in all varieties with the exception of PI481656, while it was affected by watering conditions in PI481659, PI481652, PI481644, and PI481646 (Figure 7D, Table S2). Thus, in the inflorescences, the highest flavonoid concentrations were observed under 28WW (Zlata, PI481659, PI481656, PI481371, and PI427239), 28WS (Lifago, PI481652, PI147652, PI481644, and PI481646), or both (Islek and PI481670) conditions (Figure 7D). The difference in inflorescence flavonoid concentrations among conditions ranged from 40% in Lifago to more than 70% in Islek, PI481652, and PI481670.

## 3. Discussion

We compared the resistance to water stress, high temperature, and combined stress in 12 varieties of *F. tataricum*. Our results showed that plants were more discriminated according to environmental conditions than variety, with the exception of Islek, whose development was hastened and whose plant growth and seed production were reduced compared to the other varieties. Islek is a domestic population from the Islek region of Europe (border region of Luxemburg, Germany, and Belgium) [19,28,47]. The other used varieties originated from Slovenia, Germany, United States, Nepal, and Bhutan (Table S1). A previous comparison with the Slovenian variety Zlata under field conditions also showed that Islek plants were smaller and produced fewer leaves and inflorescences [19]. Cepkova et al. compared 15 *F. tataricum* genotypes under field conditions in the Czech Republic [48], and several of them were also investigated in our study as they came from the same genebank. They reported no remarkable morphological differences among varieties and that the 1000-seed weight ranged from 8.10 to 20 g [48], which is similar to our observations. Moreover, they showed that the performance of the buckwheat varieties depended more on the weather conditions than on the variety [48], which is in accordance with our observations. Our results nevertheless showed that at 21 °C (21WW and 21WS combined), the best-performing varieties in terms of yield were PI481671 and Lifago, suggesting that these varieties could be interesting for future work.

We observed that *F. tataricum* responded differently to high temperature and water stress. High temperature increased leaf production but decreased leaf area. It also increased transpiration rate and stomatal conductance. Opening the stomata under high temperature allows decreasing the leaf temperature, and this is commonly observed in response to heat stress [49]. However, this strategy is not always relevant when heat is coupled with drought, in which case a tradeoff is needed among leaf cooling, water saving, and photosynthesis [49]. The effect of heat on chlorophyll fluorescence and net photosynthesis was less obvious in our study and depended on the variety. However, the polyphenol and flavonoid content increased with high temperature in all varieties, mainly in inflorescences. In particular, flavonoid content was high in the Lifago variety. An increase in antioxidant capacity was previously reported in *F. tataricum* under heat stress [44], and this species is known to be rich in antioxidants [19,25,48,50,51]. Antioxidants help plants respond to abiotic stress by limiting oxidative stress [3,52,53]. At the reproductive level, high temperature increased the inflorescence and flower production in *F. tataricum*. We previously observed an increase of inflorescence production in buckwheat in response to heat, although this was more marked in *F. tataricum* than in *F. esculentum* [44]. Michiyama et al. [54] also reported a prolonged flowering period and an increased number of inflorescences under high temperature in *F. esculentum*. It was proposed that, under stressful conditions, buckwheat produced more flowers to offset the lower number of flowers at anthesis [25,48,55,56]. In contrast to *F. esculentum*, *F. tataricum* self-pollinates and produces cleistogamous flowers [38]; hence, flower anthesis is less of a problem in *F. tataricum* than in *F. esculentum*. We nevertheless observed open flowers in *F. tataricum*. We also observed that high temperature hastened seed initiation in *F. tataricum* but inhibited seed development. Michiyama et al. [57] also reported that high temperature (27 °C/20 °C day/night) severely reduced seed production and yield, and that a temperature of 30 °C/23 °C day/night prevented flowering and seed set in the rice Tartary buckwheat var. Mekei T29. We previously observed that high temperature decreased pollen production and stigma receptivity in *F. tataricum* [44], which could affect ovule fertilization. However, it did not affected pollen viability [44]. In any case, we did not investigate at which growing stage seeds aborted and whether the seed abortion was due to the absence of fertilization or to problems during embryo, seed development, or seed filling. The impacts of heat stress on crop yield are the result of the integration of many processes, not all of which are affected by high temperature [12]. In general, pollen viability is highly sensitive to heat stress, followed by pistil viability, with fertilization and embryogenesis having a comparatively higher heat stress threshold [13]. However, the genus *Fagopyrum* is known for its high pollen viability, and female gametophytes were shown to be much more sensitive to high temperature than male gametophytes in buckwheat [44,58,59]. It was suggested that high temperature affects seed set more severely in Tartary buckwheat than in common buckwheat [57], but further research is requested to identify the temperature threshold that inhibits seed set in both species and the most sensitive reproductive stage. Since seed set was inhibited at 28 °C in all varieties, we could not identify a heat-resistant variety in this study.

Water stress decreased plant growth as observed by the decrease in plant height, leaf area, and plant DW. Xiao-Jiao et al. [60] and Wan et al. [43] also showed that drought stress decreased the plant height, leaf area, plant DW, and root development of *F. tataricum*. However, this contrasts with our previous results that did not show strong growth reduction by water stress in var. Zlata [45]. It has to be mentioned that we did not investigate the plant height and the leaf area in this previous study [45], and that these parameters were the most affected by water stress in this study. At the physiological level, water stress increased leaf CCI but its impact on chlorophyll fluorescence and gas exchange differed according to the varieties. Wan et al. [43] compared drought-susceptible and -resistant varieties in *F. tataricum*, and they showed that the drought-tolerant varieties maintained a higher Ai and gs but a lower Ei than the susceptible varieties in response to water stress. Xiang et al. [61] reported a positive correlation between yield and leaf CCI, Ai, and gs under rainfed conditions [61]. Enhanced rates of photosynthesis and dry matter accumulation led to higher post-anthesis accumulation of biomass with a positive impact on grain number and higher yield [61]. In our study, we observed rather a negative correlation or no correlation between physiological (Ai, CCI, Ei, and gs) and yield parameters in response to water stress (Figure S2). Water stress decreased yield parameters, although its impact was less strong than that observed for temperature increase, suggesting that *F. tataricum* is more resistant to water stress than to heat stress. Wan et al. [43] also reported a decrease in seed production in response to water stress in *F. tataricum*. Our previous comparison of the response of *F. esculentum* and *F. tataricum* to water stress showed that the former was more affected by water stress than the latter [45]. *Fagopyrum tataricum* was reported to be widely adaptable to hostile environments and resistant to abiotic stresses such as drought [41,43,62]. It is usually cultivated in the mountainous areas of western China, northern India, Bhutan, and Nepal at high altitude, and it regularly suffers from drought stress in its main growing regions [43,63]. Through whole-genome sequencing, Zhang et al. [41] investigated genes related to abiotic stress resistance (including drought) in *F. tataricum*, and their data suggest that *F. tataricum*’s ability to tolerate high levels of abiotic stress is attributed to the expansion of several gene families involved in signal transduction, gene regulation, and membrane transport. Huang et al. [62] observed also a modification of several stress-response genes and genes coding for late embryogenesis abundant (LEA) proteins in response to drought stress in *F. tataricum*, and they showed that both the ABA-dependent and the ABA-independent pathway are involved in the regulation of drought stress in this species. Our results indicated that Zlata, PI481659, and PI481670 seemed less affected by water stress than the other varieties regarding flowering and yield parameters (Table S2), although the best yield under 21 WS conditions was obtained for PI481671 and Lifago.

Our results showed that the combination of high temperature and water stress had a stronger negative impact than each stress applied separately in *F. tataricum*. The combination of both stresses was not investigated previously in this species to the best of our knowledge. Plant growth was strongly reduced under 28WS conditions, and seed development was completely inhibited. The only positive impact was the increase in polyphenol and flavonoid concentrations. An increase in antioxidant production was reported in response to the combination of heat and drought stress in other species to limit oxidative stress [64,65]. The main problem plants face during stress combination is that the two different stresses simultaneously affecting the plant could require different and sometimes opposing physiological and metabolic responses [5,9]. For example, drought and heat stress require opposing stomatal responses [5]. While plants tend to open their stomata to allow leaf cooling down under heat, they tend to close their stomata under drought to limit water loss [10]. We observed that *F. tataricum* closed their stomata under combined stress as suggested by the low Ei and gs values. A decrease in photosynthetic activity in response to the combination of heat and drought has been observed in several species including *Arabidopsis*, tobacco, soybean, tomato, maize, and wheat [5,9,10,46,66]. The reproductive stage is often more affected by stress and their combination than vegetative growth [67,68]. Pollen maturation, fertilization, embryogenesis, and seed maturation are highly sensitive to different abiotic stress conditions, such as heat and drought, which could lead to pollen, embryo or seed abortion [5,9,69]. The combination of water stress and heat exacerbates the impacts of a single stress on the reproductive processes of different crop species, directly impacting grain production [70]. It impacts plant yield by decreasing the harvest index, shortening the life cycle of crops, and altering the seed number, size, and composition [5]. We observed that, in *F. tataricum*, both stresses had an additive effect regarding yield parameters. Under combined stress, inflorescence production was decreased as observed under water stress, while the number of flowers per inflorescence was increased as observed under high temperature; seeding date was hastened as observed under high temperature, while seed set and viable seed production were inhibited as observed under high temperature. From an agronomical point of view, high temperature and combined stress are, thus, more detrimental to *F. tataricum* yield than water stress. Due to the lack of viable seed production at high temperature and under combined stress, we could not identify a resistant variety; further studies at lower temperature will be required to discriminate the most resistant variety regarding heat stress.

## 4. Materials and Methods

### 4.1. Plant Culture and Growth Conditions

Seeds of *F. tataricum* var. Zlata and var. Islek were received from Prof. Dr. Ivan Kreft (University of Ljublana, Slovenia) and Christian Zewen (Luxemburg), respectively. Seeds of the other varieties (Lifago, PI481659, PI481656, PI481652, PI427239, PI476852, PI48646, and PI481670) were obtained from the Genebank of the Crop Research Institute (CRI, Prague, Cezch Republic) (Table S1). Seeds were sown on 29 October 2019 in peat compost (DCM, Amsterdam, Netherlands) under greenhouse conditions (temperature of 22 ± 2 °C, relative humidity of 65% ± 5% and a 16 h photoperiod). In addition to natural light, supplementary lighting was provided by LED LumiGrow lights (650 W, red/blue) to maintain a 16 h photoperiod and a minimum light intensity of 150 µmol·m^−2^·s^−1^. Two weeks after sowing (November 12th), the seedlings were transplanted into 2 L pots filled with the same peat compost, and plants were grown at two temperatures (21 °C/19 °C or 28 °C/26 °C day/night) under greenhouse conditions (Figure S1A). Water stress started 3 days later; control plants were watered three times a week, while water-stressed plants were watered once or twice a week (depending on the developmental stage, in order to maintain a minimum soil water content of 15%). Soil moisture was measured every 2 days before watering to certify the well-watered condition or the water stress condition using a ProCheck sensor handheld reader (Decagon Devices, Pullman, WA, USA) (Figure S1B). Four growing conditions were, thus, compared: 21 °C well-watered (21WW), 21 °C water stress (21WS), 28 °C well-watered (28WW), and 28 °C water stress (28WS) with 10 plants per treatment and variety. Plants were maintained under these conditions until seed maturation and harvest (10 weeks after stress).

### 4.2. Plant Growth Parameters

The total number of leaves (unfolded) and inflorescences per plant were counted once a week throughout the experiment. Plant height (from the soil to the last inflorescence of the main stem) and the total number of ramifications per plant were measured at harvest. Leaf area was measured on the leaf subtending the first inflorescence on five plants per species and treatment, after 5 weeks of treatment. Leaves were scanned, and the leaf area was measured using the ImageJ software (https://imagej.nih.gov/ij/index.html, accessed on 1 April 2020).

Three plants per variety and treatment were harvested after 9 weeks of treatment for var. Islek and after 10 weeks of treatment for the other varieties. The leaves, stem, and reproductive parts were separated and weighted to determine the fresh weight (FW). The dry weight (DW) was determined after 48 h of incubation in an oven at 72 °C. The tolerance index was calculated as (tDWs − tDWc)/tDWc × 100, where tDWc is the plant DW for the 21 WW plants, and tDWs is the plant DW for stressed plants (either 21WS, 28WW, or 28WS).

### 4.3. Flowering and Seed Parameters

Flowering time was assessed by the position of the node where the first inflorescence appeared (the nodes were counted acropetally, with the cotyledonary node being disregarded). Macroscopic appearance of the first inflorescence, the first seed, and the first mature seed were recorded when, respectively, the first flower, the first green seed, and the first mature brown seed were visible, expressed as the number of days after stress.

Ten inflorescences (from the seventh or eighth nodes) were collected per variety and treatment and conserved in FAA (formaldehyde, acetic acid, and 70% alcohol, 1:1:18). The numbers of flowers, seeds, and aborted seeds per inflorescence were determined under a stereomicroscope. Seed set (%) was calculated as the number of mature seeds per inflorescence/total number of flowers (flowers + seeds + aborted seeds) per inflorescence × 100.

The number of seeds per plant and the 1000-seed weight were measured at harvest.

### 4.4. Physiological Parameters

Physiological measurements (chlorophyll fluorescence, chlorophyll content, and gas exchange) were performed on the fifth youngest expanded leaf of three plants per variety and treatment, after 8 weeks of treatment. Chlorophyll fluorescence was monitored using a fluorescence monitoring system (FMS II; Hansatech Instruments, Norfolk, United Kingdom) according to Aubert et al. [44]. The quantified parameters were photosystem II efficiency (ϕPSII) and nonphotochemical quenching (NPQ) [71]. The chlorophyll content index (CCI) was measured using a chlorophyllometer (Opti-Sciences, CCM-200), and the measurement was taken three times on the same leaf. Gas exchange was measured using an infrared gas analyzer (IRGA, ADC BioScientific LCI-SD system, Hoddesdon, United Kingdom). The temperature and relative humidity in the cuvette were set at 21 °C or 28 °C according to the growth chamber. The quantified parameters were instantaneous net photosynthesis rate (Ai), instantaneous net transpiration rate (E), and stomatal conductance (gs).

### 4.5. Polyphenol and Flavonoid Concentrations

Phenolic extracts were obtained by extraction of 100 mg of fresh frozen tissue with 80% methanol according to [44]. Total phenolic content was determined using the modified Folin–Ciocâlteu colorimetric method, and absorbances were measured at 760 nm using a spectrophotometer. The standard curve range was 0.0–800.0 μg of gallic acid·mL^−1^. Total flavonoid content was determined using the aluminum chloride chelation method. The standard curve range was 0.0–50.0 μg of quercetin·mL^−1^. The absorbance was measured at 440 nm using a spectrophotometer (UV1800, Shimadzu, ‘s-Hertogenbosh, The Netherlands).

### 4.6. Statistical Analysis

All of the analyses were conducted in R studio or SAS Enterprise Guide 8.3. The normality of the data was estimated using Shapiro–Wilk tests, and homoscedasticity was verified using Levene’s tests. The data were transformed (logarithm or square root) when required, to ensure normal distributions. The ANOVA3 models were defined to evaluate the effects of the variety, the temperature, the water stress, and their interactions on the measured parameters. ANOVA2 models were also defined to evaluate the effects of the temperature, the water stress, and their interaction on the measured parameters on each variety separately. Principal component analysis was performed to visualize the differences in plant growth, flowering, physiological, and antioxidant parameters according to the varieties and the treatments. If not indicated otherwise, data were presented as means ± SE. Differences among environmental conditions for a specific parameter (P) were calculated as (P_h_ − P_l_)/P_h_ × 100, where P_h_ is the mean value of the highest condition, and P_l_ is the mean value of the lowest condition for the same variety.

## 5. Conclusions

Heat and drought periods are expected to increase in the context of climate change; hence, it is important to study the response of crop plants to these abiotic constraints and mainly to their combination. Altogether, our study showed that Tartary buckwheat behavior differed in response to temperature increase and drought, and that the combination of the two stresses enhanced the negative effects. High temperature boosted leaf and inflorescence production, decreased leaf area, and increased antioxidant production in the inflorescences but prevented viable seed development, while drought reduced plant growth and height and increased leaf chlorophyll concentration. From an agronomical point of view, Tartary buckwheat was more affected by high temperature than by water stress as seed development was prevented at 28 °C under both well-watered and water stress conditions, strongly impacting the yield parameters. Our results also showed that plants were more discriminated according to environmental conditions than variety, which did not allow us to clearly identify resistant varieties. However, the best-performing varieties in terms of yield were PI481671 and Lifago. Moreover, Lifago was particularly rich in flavonoids, which makes it an interesting variety for further work.

## Figures and Tables

**Table 1:** Statistical results (ANOVA3) of vegetative and reproductive parameters of *F. tataricum* varieties subjected to two temperatures (21 °C vs. 28 °C) and water supply conditions (well-watered (WW) vs. water-stressed (WS)).

Parameter *	Variety	Temp	Water	Var × Temp	Var × Water	Temp × Water	Var × Temp × Water
Leaves	F = 5.58 ***	F = 454.3 ***	F = 677.6 ***	F = 14.8 ***	F = 4.8 ***	F = 501.3 ***	F = 3.8 ***
Height	F = 90.14 ***	F = 244.1 ***	F = 3246 ***	F = 2.5 **	F = 50.6 ***	F = 18.4 ***	F = 2.6 **
Leaf area	F = 53.0 ***	F = 1552.9 ***	F = 586.4 ***	F = 5.2 ***	F = 3.7 ***	F = 31.5 ***	F = 2.3 *
Ramification	F = 7.30 ***	F = 7.7 **	F = 4.8 *	F = 3.7 ***	F = 691.6 ***	F = 61.6 ***	F = 2.5 **
Tolerance index	F = 8.2 ***	F = 212.6 ***	F = 690.3 ***	F = 4.3 ***	F = 4.0 ***	F = 24.2 ***	F = 6.4 ***
Leaf DW	F = 25.7 ***	F = 1.2 NS	F = 165.6 ***	F = 1.8 *	F = 4.3 ***	F = 200.8 ***	F = 3.5 ***
Stem DW	F = 36.1 ***	F = 55.6 ***	F = 1098.4 ***	F = 3.1 **	F = 12.1 ***	F = 75.9 ***	F = 2.4 *
Inflorescences	F = 13.7 ***	F = 110.8 ***	F = 646.5 ***	F = 15.2 ***	F = 3.6 ***	F = 368.9 ***	F = 2.6 **
Node first inflo	F = 34.6 ***	F = 4.8 *	F = 0.03 NS	F = 0.9 NS	F = 0.7 NS	F = 0.9 NS	F = 2.1 *
Days to inflo	F = 55.7 ***	F = 0.3 NS	F = 22.5 ***	F = 3.6 ***	F = 3.6 ***	F = 12.0 ***	F = 2.6 **
Flowers/inflo	F = 14.3 ***	F = 203.2 ***	F = 0.02 NS	F = 17.4 ***	F = 1.9 *	F = 4.9 *	F = 1.1 NS
Days to seeds	F = 61.0 ***	F = 1611.1 ***	F = 12.6 ***	F = 13.2 ***	F = 2.3 **	F = 0.03 NS	F = 0.61 NS
Seed set	F = 4.3 ***	F = 2777 ***	F = 3.2 NS	F = 4.3 ***	F = 2.7 **	F = 3.2 NS	F = 2.7 *
Seeds/plant	F = 88.7 ***	F = 2404.5 ***	F = 1545.0 ***	F = 92.1 ***	F = 37.3 ***	F = 81.3 ***	F = 22.9 ***
1000-seed weight	F = 27.8 ***	-	F = 92.7 ***	-	F = 17.7 ***	-	-

* DW: dry weight, WC: water content, var: variety, temp: temperature, NS: not significant, * significant at 5% level, ** significant at 1% level, *** significant at 0.1% level, -: no data at 28 °C.

**Table 2:** Leaf and stem dry weight of *F. tataricum* varieties subjected to two temperatures (21 °C vs. 28 °C) and water supply conditions (well-watered (WW) vs. water-stressed (WS)).

Variety	Leaf Dry Weight (g)				Stem Dry Weight (g)			
	21WW	21WS	28WW	28WS	21WW	21WS	28WW	28WS
Islek	0.14 ± 0.01 ^ab^	0.13 ± 0.03 ^ab^	0.26 ± 0.04 ^b^	0.08 ± 0.02 ^a^	0.24 ± 0.03 ^b^	0.17 ± 0.02 ^a^	0.17 ± 0.01 ^a^	0.03 ± 0.01 ^c^
Zlata	0.68 ± 0.05 ^ab^	0.57 ± 0.05 ^ab^	1.09 ± 0.19 ^a^	0.11 ± 0.01 ^a^	1.02 ± 0.06 ^a^	0.46 ± 0.08 ^ab^	1.21 ± 0.22 ^a^	0.06 ± 0.01 ^b^
Lifago	0.49 ± 0.10 ^a^	0.71 ± 0.09 ^ab^	1.15 ± 0.24 ^b^	0.35 ± 0.02 ^a^	2.72 ± 0.28 ^b^	0.82 ± 0.11 ^a^	2.35 ± 0.17 ^b^	0.20 ± 0.01 ^c^
PI481659	0.52 ± 0.15 ^ab^	0.84 ± 0.02 ^ab^	1.22 ± 0.11 ^a^	0.37 ± 0.01 ^b^	2.33 ± 0.19 ^b^	0.82 ± 0.10 ^a^	1.95 ± 0.13 ^b^	0.22 ± 0.02 ^c^
PI481656	0.62 ± 0.05 ^a^	0.76 ± 0.08 ^b^	0.71 ± 0.11 ^b^	0.39 ± 0.06 ^a^	2.74 ± 0.08 ^b^	0.60 ± 0.08 ^a^	1.86 ± 0.05 ^d^	0.22 ± 0.04 ^c^
PI481671	1.03 ± 0.15 ^a^	0.78 ± 0.08 ^ab^	0.88 ± 0.09 ^a^	0.39 ± 0.01 ^b^	3.04 ± 0.35 ^b^	0.75 ± 0.12 ^a^	2.42 ± 0.43 ^b^	0.19 ± 0.02 ^a^
PI481652	0.45 ± 0.01 ^ab^	0.66 ± 0.03 ^a^	0.99 ± 0.05 ^c^	0.31 ± 0.09 ^b^	1.87 ± 0.3 ^b^	0.52 ± 0.03 ^a^	2.39 ± 0.3 ^b^	0.21 ± 0.03 ^a^
PI427239	1.15 ± 0.18 ^b^	0.66 ± 0.07 ^a^	1.13 ± 0.03 ^b^	0.37 ± 0.03 ^a^	2.05 ± 0.4 ^b^	0.64 ± 0.07 ^a^	2.65 ± 0.02 ^b^	0.18 ± 0.02 ^c^
PI496852	0.28 ± 0.07 ^a^	0.62 ± 0.05 ^a^	1.17 ± 0.16 ^b^	0.22 ± 0.05 ^a^	1.03 ± 0.22 ^a^	0.71 ± 0.11 ^ab^	2.09 ± 0.37 ^c^	0.18 ± 0.04 ^b^
PI481644	1.04 ± 0.10 ^a^	0.73 ± 0.05 ^ab^	1.55 ± 0.16 ^c^	0.28 ± 0.06 ^b^	3.08 ± 0.3 ^b^	0.85 ± 0.1 ^a^	2.24 ± 0.03 ^b^	0.10 ± 0.02 ^c^
PI481646	0.44 ± 0.07 ^a^	0.63 ± 0.04 ^ab^	0.91 ± 0.17 ^b^	0.31 ±0.02 ^a^	1.24 ± 0.09 ^b^	0.68 ± 0.11 ^a^	2.39 ± 0.10 ^d^	0.18 ± 0.04 ^c^
PI481670	0.69 ± 0.23 ^ab^	0.72 ± 0.09 ^ab^	1.08 ± 0.14 ^b^	0.28 ± 0.07 ^a^	2.42 ± 0.46 ^b^	0.44 ± 0.03 ^a^	2.60 ± 0.83 ^b^	0.23 ± 0.04 ^a^

Values followed by a same letter for the same variety and parameter are not statistically significant at the 5% level.

**Table 3:** Statistical results (ANOVA3) of physiological and antioxidant parameters of *F. tataricum* varieties subjected to two temperatures (21 °C vs. 28 °C) and water supply conditions (well-watered (WW) vs. water-stressed (WS)).

Parameter *	Variety	Temp	Water	Var × Temp	Var × Water	Temp × Water	Var × Temp × Water
CCI	F = 47.9 ***	F = 137.1 ***	F = 1995.7 ***	F = 7.4 ***	F = 23.8 ***	F = 133.2 ***	F = 12.2 ***
ϕPSII	F = 1.2 NS	F = 1.8 NS	F = 1.6 NS	F = 1.6 NS	F = 1.2 NS	F = 2.9 NS	F = 1.8 NS
NPQ	F = 1.1 NS	F = 14.7 ***	F = 0.2 NS	F = 1.4 NS	F = 0.9 NS	F = 1.3 NS	F = 1.1 NS
Ai	F = 1.5 NS	F = 9.8 **	F = 0.1 NS	F = 2.9 **	F = 1.9 *	F = 0.4 NS	F = 1.1 NS
Ei	F = 2.9 ***	F = 148.4 ***	F = 85.9 ***	F = 5.6 ***	F = 2.1 *	F = 73.0 ***	F = 3.7 ***
gs	F = 2.8 **	F = 28.3 ***	F = 43.4 ***	F = 5.1 ***	F = 2.5 ***	F = 46.3 ***	F = 3.8 ***
Polyphenols (leaf)	F = 14.8 ***	F = 8.4 **	F = 45.1 ***	F = 3.6 ***	F = 3.1 **	F = 1.3 NS	F = 2.0*
Polyphenols (inflo)	F = 9.3 ***	F = 783.5 ***	F = 1.5NS	F = 3.7 ***	F = 3.2 **	F = 13.2 ***	F = 6.7 ***
Flavonoids (leaf)	F = 27.3 ***	F = 6.7 *	F = 31.6 ***	F = 2.3 *	F = 3.6 ***	F = 0.4 NS	F = 1.7 NS
Flavonoids (inflo)	F = 47.9 ***	F = 420.7 ***	F = 1.2 NS	F = 4.6 ***	F = 2.6 **	F = 11.7 ***	F = 4.6 ***

* CCI: chlorophyll content index, ϕPSII: efficiency of photosystem 2, NPQ: nonphotochemical quenching, Ai: net photosynthesis rate, Ei: net transpiration rate, Ci: intercellular CO_2_ concentration, gs: stomatal conductance, inflo: inflorescence, var: variety, temp: temperature, NS: not significant, * significant at 5% level, ** significant at 1% level, *** significant at 0.1% level.

## Data Availability

The data presented in this study are available in the text and supplemental data. The data presented in this study are available on request from the corresponding author.

## Supplementary Materials

The following supporting information can be downloaded at https://www.mdpi.com/article/10.3390/plants11111517/s1: Figure S1. Environmental conditions during the experiment. (A) Temperature in the greenhouse. G1: greenhouse at 21 °C, G2: greenhouse at 28 °C. (B) Soil water content in pots before watering. Plants were grown at 21 °C or 28 °C under well-watered (WW) or water stress (WS) conditions. Watering was performed Monday, Wednesday, and Friday for WW plants and Monday and Friday for WS plants; Table S1. Name and origin of the *F. tataricum* varieties used in this study; Table S2. Statistical results (ANOVA2) of the measured parameters of *F. tataricum* varieties subjected to two temperatures (21 °C vs. 28 °C) and water supply conditions (well-watered (WW) vs. water-stressed (WS)); Figure S2. Correlations among physiological, plant growth, and yield parameters in *F. tataricum* varieties subjected to two temperatures (21 °C vs. 28 °C) and water supply conditions (well-watered vs. water-stressed). (A) Impact of temperature (21WW and 28WW), (B) impact of water stress at 21 °C (21WW and 21WS), (C) impact of water stress at 28 °C (28WW and 28WS), and (D) impact of temperature and water stress (all conditions).
