# Supplementary material for: Comparison of Heat and Drought Stress Responses among Twelve Tartary Buckwheat (Fagopyrum tataricum) Varieties
Source: Plants (Basel). 2022 Jun 6;11(11):1517. doi: 10.3390/plants11111517 (PMC9183088; doi:10.3390/plants11111517)
Supplement: Supplementary file 1 [file plants-11-01517-s001.zip › plants-1685598-supplementary.pdf]

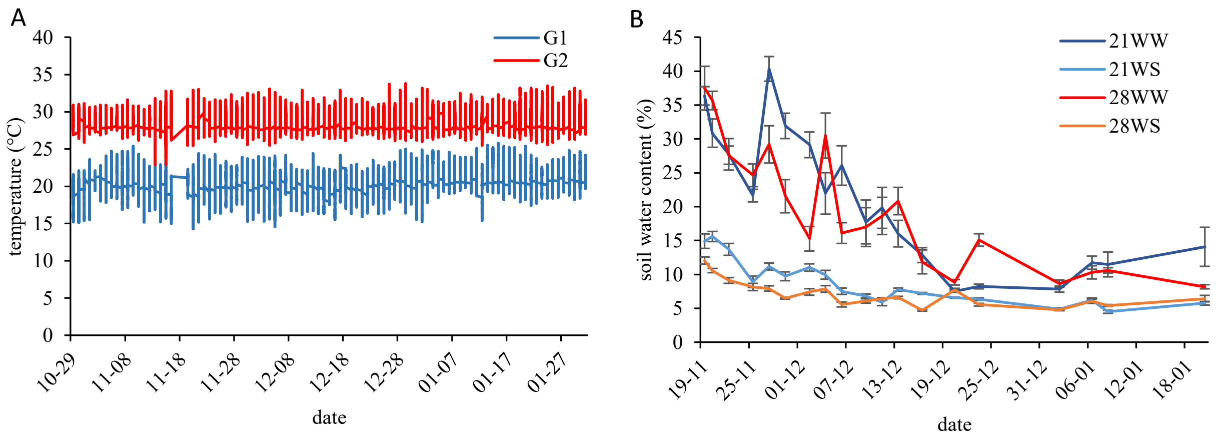

Figure S1. Environmental conditions during the experiment. (A) Temperature in the greenhouse. G1: greenhouse at 21°C, G2: greenhouse at 28°C. (B) Soil water content in pots before watering. Plants were grown at 21 °C or 28 °C under well-watered (WW) or water stress (WS) conditions. Watering was performed Monday, Wednesday, and Friday for WW plants and Monday and Friday for WS plants.

Table S1. Name and origin of the *F. tataricum* varieties used in this study.

Variety	Country of Origin	Accession Number in Genebank
Islek	LUX	/
Zlata	SLO	/
PI481644	BTN	01Z5100001
PI481656	BTN	01Z5100005
PI427239	NPL	01Z5100030
PI476852	USA	01Z5100014
PI481670	BTN	01Z5100008
Lifago	DEU	01Z5100010
PI481646	BTN	01Z5100003
PI481652	BTN	01Z5100004
PI481671	BTN	01Z5100009
PI481659	BTN	01Z5100007

Table S2. Statistical results (ANOVA2) of the measured parameters of *F. tataricum* varieties subjected to two temperatures (21°C vs. 28 °C) and water supply conditions (well-watered (WW) vs. water-stressed (WS)).

Parameter*	Islek	Zlata	Lifago	PI481659	PI481656	PI481671	PI481652	PI427239	PI476852	PI481644	PI481646	PI481670
Leaves	T: NS<br>W: NS<br>T × W: NS	T: ***<br>W: ***<br>T × W: ***	T: ***<br>W: ***<br>T × W: ***	T: NS<br>W: ***<br>T × W: **	T: NS<br>W: ***<br>T × W: ***	T: ***<br>W: ***<br>T × W: ***	T: ***<br>W: ***<br>T × W: ***	T: ***<br>W: ***<br>T × W: ***	T: ***<br>W: ***<br>T × W: ***	T: *<br>W: ***<br>T × W: ***	T: ***<br>W: ***<br>T × W: ***	T: ***<br>W: ***<br>T × W: ***
Height	T: ***<br>W: **<br>T × W: NS	T: ***<br>W: ***<br>T × W: NS	T: ***<br>W: ***<br>T × W: NS	T: ***<br>W: ***<br>T × W: **	T: ***<br>W: ***<br>T × W: ***	T: ***<br>W: ***<br>T × W: NS	T: ***<br>W: ***<br>T × W: NS	T: ***<br>W: ***<br>T × W: *	T: **<br>W: ***<br>T × W: NS	T: ***<br>W: ***<br>T × W: NS	T: ***<br>W: ***<br>T × W: NS	T: *<br>W: ***<br>T × W: *
Leaf area	T: ***<br>W: ***<br>T × W: NS	T: ***<br>W: ***<br>T × W: NS	T: ***<br>W: ***<br>T × W: NS	T: ***<br>W: ***<br>T × W: NS	T: ***<br>W: ***<br>T × W: NS	T: ***<br>W: ***<br>T × W: NS	T: ***<br>W: ***<br>T × W: NS	T: ***<br>W: ***<br>T × W: **	T: ***<br>W: ***<br>T × W: NS	T: ***<br>W: ***<br>T × W: NS	T: ***<br>W: ***<br>T × W: NS	T: ***<br>W: ***<br>T × W: NS
Ramification	T: NS<br>W: ***<br>T × W: *	T: NS<br>W: ***<br>T × W: ***	T: NS<br>W: NS<br>T × W: ***	T: NS<br>W: NS<br>T × W: NS:	T: *<br>W: ***<br>T × W: ***	T: NS<br>W: NS<br>T × W: *	T: NS<br>W: NS<br>T × W: ***	T: *<br>W: *<br>T × W: NS	T: NS<br>W: NS<br>T × W: *	T: ***<br>W: NS<br>T × W: **	T: NS<br>W: NS<br>T × W: **	T: NS<br>W: NS<br>T × W: **
Tolerance index	T: ***<br>W: *<br>T × W: ***	T: *<br>W: ***<br>T × W: NS	T: ***<br>W: ***<br>T × W: NS	T: ***<br>W: ***<br>T × W: NS	T: ***<br>W: ***<br>T × W: **	T: ***<br>W: ***<br>T × W: NS	T: ***<br>W: ***<br>T × W: NS	T: ***<br>W: ***<br>T × W: NS	T: **<br>W: ***<br>T × W: *	T: ***<br>W: ***<br>T × W: NS	T: *<br>W: ***<br>T × W: ***	T: **<br>W: ***<br>T × W: NS
Leaf DW	T: NS<br>W: **<br>T × W: *	T: NS<br>W: **<br>T × W: *	T: NS<br>W: NS<br>T × W: **	T: NS<br>W: *<br>T × W: ***	T: NS<br>W: NS<br>T × W: *	T: *<br>W: **<br>T × W: NS	T: NS<br>W: **<br>T × W: ***	T: NS<br>W: * **<br>T × W: NS	T: *<br>W: *<br>T × W: ***	T: NS<br>W: ***<br>T × W: **	T: NS<br>W: NS<br>T × W: **	T: NS<br>W: *<br>T × W: *
Stem DW	T: ***<br>W: **<br>T × W: NS	T: NS<br>W: ***<br>T × W: NS	T: *<br>W: ***<br>T × W: NS	T: **<br>W: ***<br>T × W: NS	T: NS<br>W: ***<br>T × W: NS	T: NS<br>W: ***<br>T × W: *	T: NS<br>W: ***<br>T × W: **	T: NS<br>W: ***<br>T × W: **	T: ***<br>W: ***<br>T × W: NS	T: **<br>W: ***<br>T × W: ***	T: **<br>W: ***<br>T × W: ***	T: NS<br>W: ***<br>T × W: NS
Inflorescences	T: NS<br>W: *<br>T × W: ***	T: NS<br>W: ***<br>T × W: **	T: ***<br>W: ***<br>T × W: ***	T: NS<br>W: ***<br>T × W: **	T: ***<br>W: ***<br>T × W: ***	T: ***<br>W: ***<br>T × W: ***	T: ***<br>W:***<br>T × W: ***	T: ***<br>W:***<br>T × W: ***	T: ***<br>W:***<br>T × W: ***	T: NS<br>W: ***<br>T × W: ***	T: ***<br>W:***<br>T × W: ***	T: ***<br>W:***<br>T × W: ***
Node first inflo	T: **<br>W: NS<br>T × W: **	T: NS<br>W: NS<br>T × W: NS	T: NS<br>W: **<br>T × W: NS	T: NS<br>W: NS<br>T × W: *	T: NS<br>W: NS<br>T × W: NS	T: NS<br>W: NS<br>T × W: NS	T: NS<br>W: NS<br>T × W: NS	T: NS<br>W: NS<br>T × W: NS	T: NS<br>W: NS<br>T × W: NS	T: NS<br>W: NS<br>T × W: NS	T: NS<br>W: NS<br>T × W: NS	T: NS<br>W: NS<br>T × W: NS
Days to inflo	T: NS<br>W: NS<br>T × W: NS	T: **<br>W: NS<br>T × W: NS	T: *<br>W: NS<br>T × W: NS	T: NS<br>W: NS<br>T × W: *	T: *<br>W: **<br>T × W: *	T: NS<br>W: NS<br>T × W: NS	T: **<br>W: NS<br>T × W: NS	T: ***<br>W: NS<br>T × W: NS	T: **<br>W: NS<br>T × W: NS	T: **<br>W: ***<br>T × W: *	T: NS<br>W: NS<br>T × W: NS	T: **<br>W: NS<br>T × W: NS
Flowers/inflo	T: ***<br>W: **<br>T × W: NS	T: ***<br>W: NS<br>T × W: NS	T:***<br>W: NS<br>T × W: NS	T: NS<br>W: NS<br>T × W: NS	T: *<br>W: NS<br>T × W: NS	T: *<br>W: NS<br>T × W: NS	T: ***<br>W: *<br>T × W: *	T: ***<br>W: NS<br>T × W: NS	T: ***<br>W: NS<br>T × W: NS	T: **<br>W: NS<br>T × W: NS	T: ***<br>W: *<br>T × W: NS	T: ***<br>W: NS<br>T × W: NS
Days to seeds	T: ***<br>W: NS<br>T × W: NS	T: ***<br>W: NS<br>T × W: NS	T: ***<br>W: NS<br>T × W: NS	T: ***<br>W: NS<br>T × W: NS	T: ***<br>W: *<br>T × W: NS	T: ***<br>W: NS<br>T × W: NS	T: ***<br>W: NS<br>T × W: NS	T: ***<br>W: **<br>T × W: NS	T: ***<br>W: NS<br>T × W: NS	T: ***<br>W: NS<br>T × W: NS	T: ***<br>W: ***<br>T × W: NS	T: ***<br>W: NS<br>T × W: NS
Seed set	T: ***<br>W: NS<br>T × W: NS	T: ***<br>W: NS<br>T × W: NS	T: ***<br>W: NS<br>T × W: NS	T: ***<br>W: NS<br>T × W: NS	T: ***<br>W: NS<br>T × W: NS	T: ***<br>W: NS<br>T × W: NS	T: ***<br>W: *<br>T × W: *	T: ***<br>W: NS<br>T × W: NS	T: ***<br>W: ***<br>T × W: ***	T: ***<br>W: NS<br>T × W: NS	T: ***<br>W: NS<br>T × W: NS	T: ***<br>W: NS<br>T × W: NS
Seeds/plant	T: ***<br>W: **<br>T × W: ***	T: ***<br>W: ***<br>T × W: NS	T: ***<br>W: ***<br>T × W: NS	T: ***<br>W: ***<br>T × W: NS	T: ***<br>W: ***<br>T × W: NS	T: ***<br>W: ***<br>T × W: **	T: ***<br>W: ***<br>T × W: ***	T: ***<br>W: ***<br>T × W: ***	T: ***<br>W: ***<br>T × W: ***	T: ***<br>W: ***<br>T × W: NS	T: ***<br>W: ***<br>T × W: **	T: ***<br>W: ***<br>T × W: **
1000-seed weight	W: ***	W: NS	W: ***	W: NS	W: *	W: **	W: **	W: ***	W: **	W: *	W: ***	W: NS
CCI	W: ***<br>T: ***<br>T × W: ***	W: ***<br>T: NS<br>T × W: *	W: ***<br>T: NS<br>T × W: **	W: ***<br>T: NS<br>T × W: ***	W: ***<br>T: ***<br>T × W: NS	W: ***<br>T: **<br>T × W: ***	W: ***<br>T: **<br>T × W: **	W: ***<br>T: **<br>T × W: NS	W: ***<br>T: NS<br>T × W: **	W: ***<br>T: ***<br>T × W: ***	W: ***<br>T: ***<br>T × W: NS	W: ¨***<br>T: *<br>T × W: NS
φPSII	W: NS<br>T: NS<br>T × W: NS	W: NS<br>T: NS<br>T × W: NS	W: NS<br>T: NS<br>T × W: NS:	W: NS<br>T: NS<br>T × W: NS	W: NS<br>T: NS<br>T × W: *	W: NS<br>T: NS<br>T × W: NS	W: NS<br>T: NS<br>T × W: NS	W: NS<br>T: NS<br>T × W: NS	W: NS<br>T: NS<br>T × W: NS	W: **<br>T: *<br>T × W: NS	W: NS<br>T: NS<br>T × W: *	W: NS<br>T: NS<br>T × W: NS
NPQ	W: NS<br>T: NS<br>T × W: NS	W: *<br>T: **<br>T × W: **	W: NS<br>T: NS<br>T × W: NS	W: NS<br>T: NS<br>T × W: NS	W: NS<br>T: NS<br>T × W: NS	W: NS<br>T: NS<br>T × W: NS	W: NS<br>T: *<br>T × W: NS	W: NS<br>T: NS<br>T × W: NS	W: NS<br>T: NS<br>T × W: NS	W: NS<br>T: NS<br>T × W: NS	W: NS<br>T: NS<br>T × W: NS	W: NS<br>T: ***<br>T × W: NS

Ai	T: NS<br>W: NS<br>T × W: NS	T: NS<br>W: *<br>T × W: NS	T: NS<br>W: NS<br>T × W: NS	T: NS<br>W: NS<br>T × W: NS	T: **<br>W: NS<br>T × W: NS	T: NS<br>W: NS<br>T × W: NS	T: *<br>W: NS<br>T × W: NS	T: **<br>W: NS<br>T × W: NS	T: NS<br>W: NS<br>T × W: NS	T: **<br>W: NS<br>T × W: NS	T: NS<br>W: NS<br>T × W: NS	T: NS<br>W: NS<br>T × W: NS
Ei	T: NS<br>W: ***<br>T × W: NS	T: NS<br>W: NS<br>T × W: **	T: ***<br>W: ***<br>T × W: ***	T: ***<br>W: ***<br>T × W: ***	T: ***<br>W: ***<br>T × W: ***	T: ***<br>W: **<br>T × W: *	T: ***<br>W: ***<br>T × W: ***	T: **<br>W: **<br>T × W: *	T: **<br>W: NS<br>T × W: NS	T: ***<br>W: *<br>T × W: **	T: NS<br>W: **<br>T × W: NS	T: ***<br>W: ***<br>T × W: **
gs	T: **<br>W: ***<br>T × W: NS	T: ***<br>W: ***<br>T × W: ***	T: ***<br>W: **<br>T × W: ***	T: **<br>W: **<br>T × W: ***	T: ***<br>W: NS<br>T × W: ***	T: *<br>W: *<br>T × W: NS	T: NS<br>W: *<br>T × W: **	T: *<br>W: *<br>T × W: *	T: NS<br>W: NS<br>T × W: *	T: *<br>W: NS<br>T × W: *	T: NS<br>W: **<br>T × W: NS	T: **<br>W: *<br>T × W: **
Polyphenols (leaf)	T: **<br>W: *<br>T × W: NS	T: NS<br>W: NS<br>T × W: NS	T: ***<br>W: *<br>T × W: *	T: NS<br>W: NS<br>T × W: NS	T: NS<br>W: NS<br>T × W: NS	T: NS<br>W: NS<br>T × W: NS	T: NS<br>W: NS<br>T × W: NS	T: NS<br>W: ***<br>T × W: NS	T: NS<br>W: *<br>T × W: NS	T: *<br>W: ***<br>T × W: NS	T: NS<br>W: **<br>T × W: NS	T: NS<br>W: NS<br>T × W: *
Polyphenols (inflo)	T: ***<br>W: ***<br>T × W: ***	T: ***<br>W: NS<br>T × W: *	T: ***<br>W: NS<br>T × W: NS	T: ***<br>W: *<br>T × W: ***	T: **<br>W: NS<br>T × W: NS	T: ***<br>W: NS<br>T × W: **	T: ***<br>W: NS<br>T × W:	T: **<br>W: NS<br>T × W: *	T: ***<br>W: NS<br>T × W: NS	T: ***<br>W: NS<br>T × W: NS	T: ***<br>W: NS<br>T × W: NS	T: ***<br>W: NS<br>T × W: NS
Flavonoids (leaf)	T: NS<br>W: **<br>T × W: NS	T: NS<br>W: NS<br>T × W: NS	T: **<br>W: NS<br>T × W: NS	T: NS<br>W: NS<br>T × W: NS	T: NS<br>W: NS<br>T × W: NS	T: NS<br>W: NS<br>T × W: NS	T: NS<br>W: NS<br>T × W: NS	T: NS<br>W: *<br>T × W: NS	T: NS<br>W: NS<br>T × W: NS	T: NS<br>W: ***<br>T × W: NS	T: NS<br>W: **<br>T × W: NS	T: NS<br>W: NS<br>T × W: *
Flavonoids (inflo)	T: ***<br>W: NS<br>T × W: NS	T: **<br>W: NS<br>T × W: NS	T: ***<br>W: NS<br>T × W: *	T: **<br>W: **<br>T × W: ***	T: NS<br>W: NS<br>T × W: *	T: **<br>W: NS<br>T × W: *	T: ***<br>W: **<br>T × W: *	T: ***<br>W: NS<br>T × W: NS	T: **<br>W: NS<br>T × W: NS	T: ***<br>W: *<br>T × W: NS	T: ***<br>W: *<br>T × W: NS	T: ***<br>W: NS<br>T × W: NS

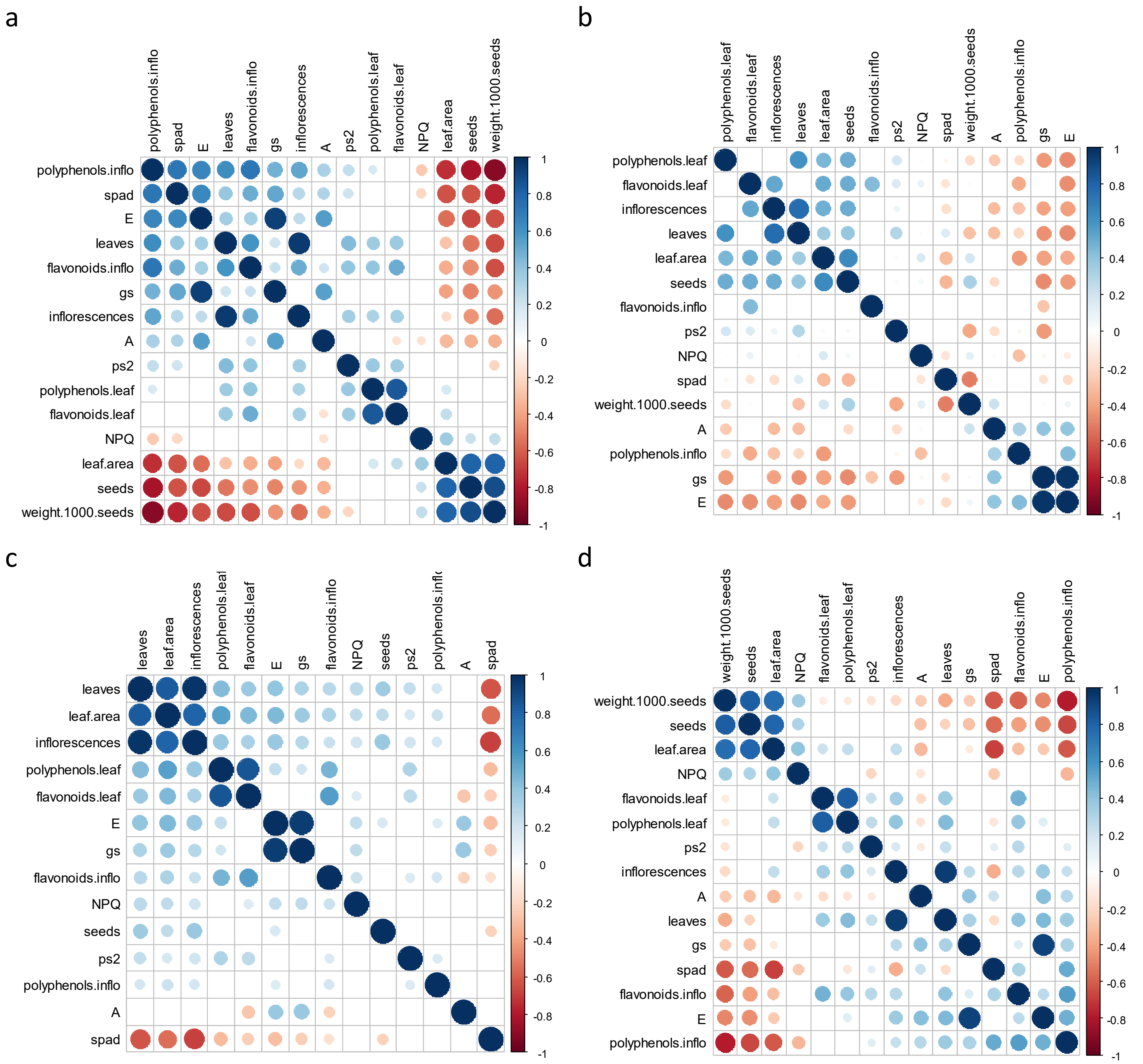

Figure S2. Correlations among physiological, plant growth, and yield parameters in *F. tataricum* varieties subjected to two temperatures (21 °C vs. 28 °C) and water supply conditions (well-watered vs. water-stressed). (a) Impact of temperature (21WW and 28WW), (b) impact of water stress at 21 °C (21WW and 21WS), (c) impact of water stress at 28 °C (28WW and 28WS), and (d) impact of temperature and water stress (all conditions).
